# Cyberbullying and Mental Health in Adults: The Moderating Role of Social Media Use and Gender

**DOI:** 10.3389/fpsyt.2021.674298

**Published:** 2021-07-15

**Authors:** Kaitlyn B. Schodt, Selena I. Quiroz, Brittany Wheeler, Deborah L. Hall, Yasin N. Silva

**Affiliations:** ^1^Department of Psychology, University of New Mexico, Albuquerque, NM, United States; ^2^School of Social and Behavioral Sciences, Arizona State University, Glendale, AZ, United States; ^3^Department of Psychology, Arizona State University, Tempe, AZ, United States; ^4^School of Mathematical and Natural Sciences, Arizona State University, Glendale, AZ, United States

**Keywords:** cyberbullying and cyber aggression, mental health, social media use, gender, adults, depression, anxiety, substance use

## Abstract

Previous research has identified a link between mental health and cyberbullying, primarily in studies of youth. Fewer studies have examined cyberbullying in adults or how the relation between mental health and cyberbullying might vary based on an individual's social media use. The present research examined how three indicators of mental health—depression, anxiety, and substance use—interact with social media use and gender to predict cyberbullying in adults. In Study 1, U.S. adults recruited through Amazon Mechanical Turk (*N* = 525) completed an online survey that included measures of mental health and cyberbullying. Multiple regression analyses revealed significant three-way interactions between mental health, degree of social media use, and gender in models predicting cyberbullying victimization and perpetration. Specifically, for men, depression and anxiety predicted greater cyberbullying victimization and perpetration, particularly among men with relatively higher levels of social media use. In contrast, depression and anxiety were uncorrelated with cyberbullying for women, regardless of level of social media use. Study 2 largely replicated these findings using well-validated measures of mental health (e.g., Center for Epidemiological Studies-Depression scale, Beck Anxiety Inventory, Global Appraisal of Individual Needs Substance Use scale) in U.S. adults recruited through Prolific.co (*N* = 482). Together, these results underscore the importance of examining mental health correlates of cyberbullying within the context of social media use and gender and shed light on conditions in which indicators of mental health may be especially beneficial for predicting cyberbullying in adults.

## Introduction

A recent survey by the Pew Research Center found that 59% of U.S. teens have experienced cyberbullying (1), highlighting a considerable rise in cyberbullying in the past several years. This increase has occurred in tandem with the widespread use of social media across broad segments of the population (2, 3) and is not limited to youth. That is, although the majority of empirical studies on cyberbullying have focused on children and teens (4), cyberbullying and related phenomena (e.g., online harassment, cyberaggression, cyberincivility, toxic social media interactions) are a problem among adults as well (5–7).

A better understanding of cyberbullying—frequently defined as intentionally harmful behavior that occurs repeatedly over time *via* electronic media [e.g., (8)]—is crucial in light of the well-documented link between cyberbullying and mental health. Specifically, cyberbullying victimization (CBV) has been associated with increased depression, anxiety, and substance use in adults (6, 9–11) and cyberbullying perpetration (CBP) has been linked with increased depression and substance use (11). These findings are consistent with meta-analyses identifying a reliable correlation between cyberbullying and mental health in children and teens [e.g., (12, 13)]. In a meta-analysis by Kowalski et al. (13), for example, CBV was correlated with greater depression, anxiety, drug and alcohol problems, and suicidal ideation among youth, and CBP was correlated with greater depression, anxiety, and substance use.

There are, however, key limitations of the existing research on cyberbullying and mental health. First, empirical investigations of cyberbullying have focused almost exclusively on children and teens [see (14), for a review]. There has been comparably less research investigating cyberbullying among adults and, of these studies, the vast majority have either examined cyberbullying among college students [see (15)] or adults in workplace settings (16, 17) [see also (4)]. Research investigating cyberbullying among adults in the general population is scarce, with even fewer studies examining the link between cyberbullying and mental health in adults. Yet, cyberbullying may manifest differently in and have a differential impact on adult vs. youth populations (9, 18, 19). This may especially be the case for cyberbullying on social media, given differences in degree of social media use and the use of specific social media platforms between teens and adults (20). Cyberbullying in adults thus remains an important and understudied phenomenon.

Second, the extant literature on cyberbullying has primarily examined bivariate correlations between cyberbullying and psychological variables—which are reported as risk factors for or outcomes associated with cyberbullying [e.g., (13)]—rather than how mental health and people's broader motives, attitudes, and behaviors may interactively predict cyberbullying. As a result, relatively little is known about the circumstances in which indicators of mental health are especially strong correlates of CBV or CBP. We argue that the relation between mental health and cyberbullying might best be understood within the context of individuals' social media use. In particular, we propose that factors pertaining to an individual's social media use may moderate the association between cyberbullying and mental health.

One possibility is that the correlation between cyberbullying and mental health may be especially strong among individuals with greater social media use. This is supported by research indicating that social media use corresponds with an increased likelihood of cyberbullying (21, 22) and, when used in certain ways, is linked with poorer mental health (23–25). According to routine activity theory (26), for example, CBV is most likely to occur when cyberbullying victims and perpetrators use the same online spaces (e.g., social media platforms) without effective safeguards (e.g., platform policies, moderators, privacy settings) in place. This framework highlights the increased opportunity for cyberbullying that comes with higher degrees of social media use.

A number of studies have also found that social media use is predictive of poorer mental health. Meta-analyses, for instance, have revealed a small but statistically significant positive correlation between degree of daily social media use and depression (19, 27), with evidence of a stronger link in adult compared to adolescent samples [(19); see also (28–30)]. Greater daily social media use has also been associated with heightened dispositional anxiety and an increased likelihood of meeting the clinical criteria for an anxiety disorder (31).

Finally, because gender differences in cyberbullying experiences (7, 32–34), social media use (35), and mental health (36, 37) have emerged in previous research, the extent to which mental health and social media use interactively predict CBV and CBP might vary systematically between men and women. Whereas some studies have found higher rates of both CBV and CBP in men than women (32, 33), others have found CBV to be more prevalent among women (7, 34). Interestingly, Wang et al. (7) found that CBV was more prevalent among women than men, but only when considering lifetime history of cyberbullying; there were no gender differences in CBV prevalence rates within the past month. It is thus worth noting that gender differences in cyberbullying have varied considerably across the literature.

Particularly relevant are studies investigating gender differences in the relation between cyberbullying and mental health. Several studies have found a stronger association between cyberbullying and mental health among women (and girls) compared to men (and boys) [(38, 39); see also (12, 13)]. Painting a more nuanced picture of how gender moderates the cyberbullying-mental health link, however, (40) found that CBV in adolescents was more strongly associated with emotional problems (i.e., depression, anxiety) for females and more strongly associated with behavioral problems (e.g., conduct disorder) for males. In light of additional complexity in the nature of gender differences in social media use and the prevalence of specific psychological disorders, investigating gender in the context of the interrelations among cyberbullying, social media use, and mental health may be particularly beneficial.

In sum, a vital question that has yet to receive sufficient empirical attention is how the relation between cyberbullying and mental health in adults might vary as a function of social media use. The primary aim of the present research was thus to examine how three indicators of mental health—depression, anxiety, and substance use—interact with social media use factors to predict CBV and CBP. Evidence of such an interaction would shed light on conditions under which indicators of mental health are more vs. less beneficial for predicting cyberbullying. Our second aim was to contribute to the relatively scarce literature on cyberbullying and mental health among adults. To this end, we recruited adult samples with a broad range of ages to complement existing work on cyberbullying among college populations and assessed general cyberbullying experiences to complement existing research on cyberbullying in the workplace. Our third aim was to investigate gender differences in the interrelations among mental health, social media use, and cyberbullying. Although some research has identified a stronger link between cyberbullying and mental health for women, these findings are far from unequivocal and may be further shaped by gender differences in social media use and specific psychological disorders. Therefore, we explored the possibility that gender moderates the potential interactive effect of mental health and social media use on cyberbullying. We tested these research questions in two studies with adults in the U.S. recruited from online survey platforms.

## Study 1

As an initial test of the extent to which mental health correlates of cyberbullying vary as a function of degree of social media use, we analyzed data collected as part of a broader study on sociodemographic, psychological, and social media use predictors of cyberbullying victimization and perpetration in adults (41).

### Materials and Methods

Participants took a 15-min online survey that included measures of CBV and CBP, individuals' history of depression, anxiety, and substance use, and frequency of social media use. IRB approval was obtained prior to data collection.

#### Participants

Data were collected from a sample of 530 U.S. adults through Amazon Mechanical Turk, a crowdsourcing platform commonly used by researchers in the social sciences (42, 43). Given the central role of gender in our analyses, participants who indicated *prefer not to answer* for gender (*n* = 3) or who had missing data for gender (*n* = 2) were excluded, resulting in a final sample size of 525 (see Table 1 for demographic information).

**Table 1:** Study 1: participant demographics.

***N***		**525**
**Age**		
	Mean	36.7
	Standard deviation	12.7
	Range	18–89
**Gender**		
	Men	269 (51.2%)
	Women	256 (48.8%)
**Sexual orientation**		
	Heterosexual	442 (84.2%)
	Gay or lesbian	26 (5.0%)
	Bisexual	47 (9.0%)
	Other	2 (0.4%)
	Prefer not to answer	3 (0.6%)
	Missing	5 (1.0%)
**Race**		
	White or European American	368 (70.1%)
	Black or African American	61 (11.6%)
	Hispanic or Latino	40 (7.6%)
	Asian or Asian American	36 (6.9%)
	American Indian or Alaska Native	2 (0.4%)
	Multiracial	17 (3.2%)
	Other	1 (0.2%)
**Education level**		
	Some high school	3 (0.6%)
	High school degree or equivalent	41 (7.8%)
	Some college, without degree	124 (23.6%)
	Associate's (2-year degree)	59 (11.2%)
	Bachelor's (4-year degree)	220 (41.9%)
	Graduate degree	76 (14.5%)
	Missing	2 (0.4%)
**Annual household income**		
	< $25,000	94 (17.9%)
	$25,000–34,999	84 (16.0%)
	$35,000–49,999	81 (15.4%)
	$50,000–74,999	129 (24.6%)
	$75,000–99,999	70 (13.3%)
	$100,000–149,999	36 (6.9%)
	$150,000–199,999	23 (4.4%)
	$200,000 or more	7 (1.3%)
	Missing	1 (0.2%)

#### Materials

##### Mental Health

Participants reported the extent to which they have experienced depression, anxiety, and substance use in separate questions. Specifically, participants were asked: (1) “To what extent have you experienced depression in the past?”; (2) To what extent have you experienced anxiety in the past?”; (3) and “To what extent have you experienced a problem with substance use in the past?”. Responses were measured along a 5-point response scale from *not at all* to *a great extent*.

##### Social Media Use

Participants were asked (1) how many hours per day they spend, on average, using social media, and (2) how often they post status updates on the social media platform they use most frequently, with responses for the latter item measured on a 6-point scale from *never* to *several times a day*.

##### Cyberbullying

We measured cyberbullying experiences using items adapted from previous research (44). To assess CBV, participants indicated how often seven types of online incidents had happened to them in the past 2 months: (1) received threatening or aggressive comments on social media; (2) received rude or nasty comments from someone else on social media; (3) was the target of rumors spread online, whether they were true or not; (4) received a mean or hurtful video/picture; (5) someone intentionally shared an embarrassing picture or video of you in order to tease or hurt you; (6) someone pretended to be you online in order to tease or hurt you; (7) someone posted pictures of you online in order to tease or hurt you. Response options included *never, only 1 or 2 times, 3–6 times, 7–8 times*, and *more than 8 times*. The items (α = 0.95) were averaged to create a composite CBV variable, with higher scores indicating higher levels of cyberbullying victimization. To assess CBP, participants indicated how often they had performed six cyberbullying behaviors in the past 2 months: (1) made rude or nasty comments to someone on social media; (2) spread rumors about others on social media; (3) sent threatening or aggressive comments while online; (4) posted a mean or hurtful video/picture of someone; (5) teased someone electronically; (6) used information found online to tease or embarrass others,” with response options including *never, only 1 or 2 times, 3–6 times, 7–8 times*, and *more than 8 times*. These items (α = 0.96) were averaged to create a composite CBP variable, with higher scores indicating higher levels of cyberbullying perpetration.

#### Analytic Strategy

We investigated the extent to which social media use moderates the relation between mental health and cyberbullying by performing a series of multiple regressions using the PROCESS macro [v.3.5 (45)] for SPSS. In each regression model, one mental health variable (i.e., depression, anxiety, substance use), one social media use variable (i.e., hours of daily social media use, frequency of status of updates), a grouping variable for gender (coded: men = −1, women = 1), and all potential interactions were entered as predictors of either CBV or CBP. Missing data for single-item indicators (i.e., age, mental health variables) were deleted pairwise, yielding sample sizes from 493 to 521 for specific models. Composite scores for the multi-item scales (i.e., CBV, CBP) reflect the mean of available items for cases with item-level missing data. Continuous predictors were mean-centered prior to the calculation of interaction terms and significant interactions were probed at 1 *SD* below the mean, at the mean, and at 1 *SD* above the mean for a given moderator. Additionally, participants' age was included in all models as a covariate.

### Results

Descriptive statistics and bivariate correlations are reported in Table 2. Below, we summarize the primary results of the analyses with each mental health variable, hours of daily social media use, and gender as predictors of CBV and CBP. The main and interaction effects for all models tested, including the models with frequency of status updates as the social media use variable, are presented in Table 3.

**Table 2:** Study 1: descriptives and bivariate correlations for major study variables.

		***M***	***SD***	**1**	**2**	**3**	**4**	**5**	**6**	**7**	**8**
1	Depression	2.75	1.28	–							
2	Anxiety	2.98	1.34	0.75^***^	–						
3	Substance use	1.92	1.24	0.32^***^	0.30^***^	–					
4	Social media use	2.99	2.92	−0.01	−0.04	0.10^*^	–				
5	Frequency of status updates	3.08	1.41	0.05	0.09^+^	0.11^*^	0.30^***^	–			
6	Cyberbullying victimization	1.61	0.92	0.02	0.02	0.35^***^	0.31^***^	0.31^***^	–		
7	Cyberbullying perpetration	1.49	0.90	0.01	0.00	0.36^***^	0.29^***^	0.26^***^	0.88^***^	–	
8	Age	36.65	12.70	−0.18^***^	−0.22^***^	−0.15^**^	−0.12^**^	−0.10^*^	−0.14^**^	−0.14^**^	–
9	Gender	–	–	0.05	0.17^***^	−0.14^**^	0.06	0.01	−0.17^***^	−0.18^***^	0.13^**^

*Gender (men = −1, women = +1)*;

*** *p < 0.001*;

** *p < 0.01*;

* *p < 0.05*;

+ *p = 0.05*.

**Table 3:** Study 1: results of regression analyses.

	**Cyberbullying victimization**				**Cyberbullying perpetration**			
**Daily social media use (in hours)**	***b***	***SE***	***t***	***R*^**2**^**	***b***	***SE***	***t***	***R*^**2**^**
***Depression (N=495)***				0.20				0.17
Age	−0.00	0.00	−1.49		−0.00	0.00	−1.56	
Depression	0.05^+^	0.03	1.71		0.03	0.03	1.12	
Daily social media use	0.13^***^	0.01	8.87		0.11^***^	0.01	8.02	
Gender	−0.19^***^	0.04	−5.14		−0.19^***^	0.04	−5.11	
Depression × daily SM use	0.05^***^	0.01	4.70		0.05^***^	0.01	4.27	
Depression × gender	−0.09^**^	0.03	−3.18		−0.07^*^	0.03	−2.43	
Daily SM use × gender	−0.06^***^	0.01	−3.96		−0.04^**^	0.01	−2.80	
Depression × daily SM use × gender	−0.03^**^	0.01	−3.11		−0.03^**^	0.01	−3.07	
***Anxiety (N=493)***				0.22				0.20
Age	−0.00	0.00	−1.50		−0.01^+^	0.00	−1.69	
Anxiety	0.09^**^	0.03	3.12		0.07^*^	0.03	2.40	
Daily social media use	0.15^***^	0.02	9.51		0.13^***^	0.02	8.71	
Gender	−0.22^***^	0.04	−5.94		−0.22^***^	0.04	−5.90	
Anxiety × daily SM use	0.06^***^	0.01	−5.47		0.06^***^	0.01	5.61	
Anxiety × gender	−0.11^***^	0.03	−4.04		−0.09^***^	0.03	−3.37	
Daily SM use × gender	−0.09^***^	0.02	−5.80		−0.07^***^	0.02	−4.88	
Anxiety × daily SM use × gender	−0.05^***^	0.01	−4.22		−0.05^***^	0.01	−4.14	
***Substance use (N=495)***				0.27				0.29
Age	−0.00	0.00	−1.11		−0.00	0.00	−1.35	
Substance use	0.21^***^	0.03	7.05		0.21^***^	0.03	7.57	
Daily social media use	0.09^***^	0.01	6.79		0.07^***^	0.01	5.74	
Gender	−0.14^***^	0.04	−3.99		−0.14^***^	0.04	−4.07	
Substance use × daily SM use	0.04^***^	0.01	4.22		0.06^***^	0.01	6.20	
Substance use × gender	−0.08^**^	0.03	−2.77		−0.07^**^	0.03	−2.63	
Daily SM use × gender	−0.03^*^	0.01	−2.49		−0.02	0.01	−1.44	
Substance use × daily SM use × gender	−0.00	0.01	−0.28		−0.01	0.01	−0.81	
**Frequency of status updates**								
***Depression (N=521)***				0.15				0.11
Age	−0.01^*^	0.00	−2.34		−0.01^*^	0.00	−2.27	
Depression	0.00	0.03	0.17		−0.00	0.03	−0.09	
Freq of status updates	0.19^***^	0.03	6.98		0.16^***^	0.03	5.90	
Gender	−0.14^***^	0.04	−3.60		−0.14^***^	0.04	−3.75	
Depression × freq status updates	0.04^+^	0.02	1.79		0.03	0.02	1.39	
Depression × gender	−0.06^+^	0.03	−1.96		−0.04	0.03	−1.33	
Freq of status updates × gender	−0.04	0.03	−1.41		−0.02	0.03	−0.74	
Depression × freq status updates × gender	−0.03	0.02	−1.51		−0.02	0.02	−1.14	
***Anxiety (N=519)***				0.15				0.12
Age	−0.01^**^	0.00	−2.73		−0.01^**^	0.00	−2.78	
Anxiety	0.00	0.03	0.14		−0.01	0.03	−0.21	
Freq of status updates	0.19^***^	0.03	6.78		0.15^***^	0.03	5.53	
Gender	−0.13^***^	0.04	−3.45		−0.14^***^	0.04	−3.66	
Anxiety × freq status updates	0.04^+^	0.02	1.89		0.04^+^	0.02	1.82	
Anxiety × gender	−0.06^*^	0.03	−2.05		−0.04	0.03	−1.43	
Freq of status updates × gender	−0.05^+^	0.03	−1.68		−0.03	0.03	−1.19	
Anxiety × freq status updates × gender	−0.04^*^	0.02	−2.24		−0.02	0.02	−1.26	
***Substance use (N=521)***				0.24				0.22
Age	−0.00	0.00	−1.24		−0.00	0.00	−1.29	
Substance use	0.21^***^	0.03	7.24		0.22^***^	0.03	7.61	
Freq of status updates	0.17^***^	0.03	6.41		0.13^***^	0.03	5.25	
Gender	−0.10^**^	0.04	−2.77		−0.11^**^	0.04	−2.95	
Substance use × freq status updates	0.05^**^	0.02	2.69		0.07^***^	0.02	3.33	
Substance use × gender	−0.07^*^	0.03	−2.29		−0.06^+^	0.03	−1.90	
Freq of status updates × gender	−0.02	0.03	−0.92		−0.01	0.03	−0.20	
Substance use × freq status updates × gender	−0.04^*^	0.02	−2.02		−0.03	0.02	−1.53	

*This table includes the unstandardized regression coefficients, associated standard errors and t-values, and overall model R^2^ values for each main regression analysis, organized by dependent variable (CBV or CBP). The top three panels report the results with daily social media use (Daily SM Use). The lower three panels report the results with frequency of status updates (Freq Status Updates). Gender was coded: men = −1, women = 1*;

*** *p < 0.001*;

** *p < 0.01*;

* *p < 0.05*;

+ *p < 0.10*.

#### Depression

In the model predicting CBV from depression, daily social media use, and gender, there was a significant three-way interaction, *b* = −0.03, *SE* = 0.01, *t*_(486)_ = −3.11, *p* = 0.002, that was driven by a significant Depression × Daily Social Media Use interaction, *b* = 0.09, *F*_(1, 486)_ = 19.73, *p* < 0.0001, that emerged for men only. The nature of this two-way interaction was such that, for men with relatively lower levels of daily social media use (1 *SD* below the mean), a greater history of depression was a marginally significant predictor of lower CBV, *b* = −0.10, *SE* = 0.06, *t*_(486)_ = −1.71, *p* = 0.087. There was, however, a significant positive correlation between depression and CBV at moderate (mean), *b* = 0.14, *SE* = 0.04, *t*_(486)_ = 3.30, *p* = 0.001, and relatively higher levels (1 *SD* above the mean) of daily social media use, *b* = 0.39, *SE* = 0.08, *t*_(486)_ = 4.91, *p* < 0.0001. In contrast, for women, the relation between depression and CBV was non-significant across all levels of daily social media use.

In the model predicting CBP from depression, daily social media use, and gender, we found a significant three-way interaction, *b* = −0.03, *SE* = 0.01, *t*_(486)_ = −3.07, *p* = 0.002, that was, once again, driven by a significant Depression × Daily Social Media Use interaction, *b* = 0.08, *F*_(1, 486)_ = 17.37, *p* < 0.0001, that emerged for men only. For men with lower daily social media use, a greater history of depression was associated with lower CBP, *b* = −0.13, *SE* = 0.06, *t*_(486)_ = −2.11, *p* = 0.035. The relation between depression and CBP was positive at moderate, *b* = 0.10, *SE* = 0.04, *t*_(486)_ = 2.40, *p* = 0.017, and higher levels of daily social media use, *b* = 0.34, *SE* = 0.08, *t*_(486)_ = 4.22, *p* < 0.0001. For women, depression and CBP were unrelated across all levels of daily social media use.

#### Anxiety

In the model predicting CBV from anxiety, daily social media use, and gender, a significant three-way interaction emerged, *b* = −0.05, *SE* = 0.01, *t*_(484)_ = −4.22, *p* < 0.0001, driven by a significant Anxiety × Daily Social Media Use interaction, *b* = 0.11, *F*_(1, 484)_ = 30.72, *p* < 0.0001, for men only. For men with relatively lower daily social media use (−1 *SD*), a marginally significant negative relation between anxiety and CBV emerged, *b* = −0.10, *SE* = 0.06, *t*_(484)_ = −1.78, *p* = 0.076, with greater anxiety corresponding with lower CBV. For men with moderate, *b* = 0.20, *SE* = 0.04, *t*_(484)_ = 4.83, *p* < 0.0001, and relatively higher levels of daily social media use, *b* = 0.51, *SE* = 0.08, *t*_(484)_ = 6.46, *p* < 0.0001, a greater history of anxiety predicted greater CBV. The relation between anxiety and CBV was non-significant across all levels of daily social media use for women.

In the model predicting CBP from anxiety, daily social media use, and gender, there was a significant three-way interaction, *b* = −0.05, *SE* = 0.01, *t*_(484)_ = −4.14, *p* < 0.0001, driven by a significant Anxiety × Daily Social Media Use interaction, *b* = 0.11, *F*_(1, 484)_ = 31.09, *p* < 0.0001, that emerged for men only. For men with lower daily social media use, greater anxiety was a significant predictor of lower levels of CBP, *b* = −0.15, *SE* = 0.06, *t*_(484)_ = −2.48, *p* = 0.013. The correlation between anxiety and CBP was positive and significant at moderate, *b* = 0.16, *SE* = 0.04, *t*_(484)_ = 3.89, *p* = 0.0001, and higher levels of daily social media use, *b* = 0.47, *SE* = 0.08, *t*_(484)_ = 5.98, *p* < 0.0001. For women, anxiety and CBP were unrelated across all levels of daily social media use.

#### Substance Use

In the model predicting CBV from substance use, daily social media use, and gender, significant two-way interactions between substance use and daily social media use, *b* = 0.04, *SE* = 0.01, *t*_(486)_ = 4.22, *p* < 0.0001, between substance use and gender, *b* = −0.08, *SE* = 0.03, *t*_(486)_ = −2.77, *p* = 0.006, and between gender and daily social media use, *b* = −0.03, *SE* = 0.01, *t*_(486)_ = −2.49, *p* = 0.013, emerged. Notably, the three-way interaction was non-significant. In light of this, we tested an additional regression model with substance use, daily social media use, gender, the Substance Use × Daily Social Media Use interaction term, and the Substance Use × Gender interaction term as predictors of CBV with age as a covariate. The results of this follow-up analysis revealed that the conditional effect of substance use on CBV was significantly stronger for men than for women and significantly stronger for participants with relatively higher levels of daily social media use.

In the model predicting CBP from substance use, daily social media use, and gender, significant two-way interactions between substance use and daily social media use, *b* = 0.06, *SE* = 0.01, *t*_(486)_ = 6.20, *p* < 0.0001, and between substance use and gender emerged, *b* = −0.07, *SE* = 0.03, *t*_(486)_ = −2.63, *p* = 0.009. Because the three-way interaction was non-significant, we once again performed a follow-up regression analysis with substance use, daily social media use, gender, the two-way interaction between substance use and daily social media use, and the two-way interaction between substance use and gender entered as predictors of CBP with age covaried. The results indicated that the conditional effect of substance use on CBP was significantly stronger for men and for participants with greater daily social media use.

### Discussion

Study 1 provided an initial test of the interaction of mental health and social media use in predicting CBV and CBP in adults. Counter to previous research documenting a stronger link between cyberbullying and mental health among women (and girls), CBV and CBP were largely uncorrelated with depression, anxiety, and substance use for the women in our sample. A consistent link between cyberbullying and mental health did, however, emerge for the men in our sample. Specifically, men with higher levels of depression, anxiety, and substance use reported greater CBV and CBP, and these effects tended to be stronger among men who indicated greater daily social media use.

Although Study 1 offered preliminary evidence of an interaction between mental health and social media use in the prediction of cyberbullying for men, it also had several crucial limitations. Foremost, we relied on single-item measures of depression, anxiety, and substance use rather than established multi-item scales. The inclusion of the single-item mental health indicators in a broader data collection effort provided a convenient opportunity to explore the effects of interest in the present research. The reliability and generalizability of the findings, however, are limited due to this methodological feature. Relatedly, these items assessed the extent of individuals' *history* of depression, anxiety, and substance use, which may or may not be reflective of one's current mental health status.

Moreover, we measured social media use by asking participants how many hours they typically spend on social media in a day and the frequency with which they post status updates. Given ambiguity in the wording of the first item, participants may have been unclear on whether to report the number of hours they are active on social media or the number of hours they are logged into a social media account on a computer or mobile device. The question about status update frequency more clearly assessed active social media use, yet status updates are just one way that individuals may be active on social media. They may, for instance, be active by communicating with others *via* direct message or posting content on other people's pages, without necessarily posting status updates themselves.

Finally, previous research has found a positive correlation between CBV and CBP—one that tends to be stronger than the overlap between traditional (face-to-face) bullying victimization and perpetration [see (46)]. To illustrate, in the meta-analysis by Kowalski et al. (13), the strongest predictor of CBP among youth—averaging across 91 independent studies—was history of CBV. Notably, however, the correlation between CBV and CBP in Study 1 (*r* = 0.88) was considerably larger than the average effect identified by Kowalski et al. (of *r* = 0.51). It is thus likely that the parallel results of our regression models predicting CBV and CBP stemmed from this unexpectedly high degree of overlap.

## Study 2

In Study 2, we sought to replicate the primary findings from Study 1 using well-validated, multi-item scales to assess current symptoms of depression, anxiety, and substance use and to more clearly distinguish active vs. more passive forms of social media use.

### Materials and Methods

#### Participants

A sample of 504 U.S. adults completed a 10–12 min survey through the online survey platform, Prolific.co. Participants who took <5 min to complete the survey (*n* = 8) or who failed both of two attention checks (*n* = 1) were excluded, as were participants who indicated *prefer not to answer* (*n* = 4), *other* (*n* = 6), or *transgender* (*n* = 3) for gender, resulting in a final sample of *N* = 482 (see Table 4 for demographic information).

**Table 4:** Study 2: participant demographics.

***N***		**482**
**Age**		
	Mean	30.03
	Standard deviation	11.57
	Range	18–79
**Gender**		
	Men	207 (42.9%)
	Women	275 (57.1%)
**Sexual orientation**		
	Heterosexual	375 (77.8%)
	Gay or Lesbian	25 (5.2%)
	Bisexual	61 (12.7%)
	Questioning	7 (1.5%)
	Other	8 (1.7%)
	Prefer not to answer	4 (0.8%)
	Missing	2 (0.4%)
**Race**		
	White	318 (66.0%)
	Black/African American	53 (11.0%)
	Hispanic/Latinx	56 (11.6%)
	Asian or Asian American	73 (15.1%)
	American Indian or Alaska Native	6 (1.2%)
	Native Hawaiian or Pacific Islander	1 (0.2%)
	Multiracial	10 (2.1%)
	Other	3 (0.6%)
	Prefer not to answer	1 (0.2%)
**Education level**		
	Some high school	5 (1.0%)
	High school degree or equivalent	42 (8.7%)
	Some college, without degree	152 (31.5%)
	Associate's (2-year degree)	44 (9.1%)
	Bachelor's (4-year degree)	170 (35.3%)
	Graduate degree	65 (13.5%)
	Other	3 (0.6%)
	Missing	1 (0.2%)
**Annual household income**		
	< $25,000	102 (21.2%)
	$25,000–34,999	58 (12.0%)
	$35,000–49,999	77 (16.0%)
	$50,000–74,999	90 (18.7)%
	$75,000–99,999	51 (10.6%)
	$100,000–149,999	65 (13.5%)
	$150,000–199,999	19 (3.9%)
	$200,000 or more	20 (4.1%)

#### Materials

##### Mental Health

We used well-validated multi-item measures of depression, anxiety, and substance use, with the order of these three scales randomized between participants.

*Depression*. We administered the 20-item Center for Epidemiological Studies-Depression scale (CES-D) (47), which asks participants how frequently they have experienced a range of symptoms of depression (e.g., felt depressed, did not feel like eating, had crying spells, talked less than usual) in the past week. Responses were measured on a 4-point scale from (1) *rarely or none of the time (*<*1 day)* to (4) *most or all the time (5–7 days)*. After reverse-scoring the appropriate items, participants' responses were averaged to create a composite depression score (α = 0.93).

*Anxiety*. We administered the 21-item Beck Anxiety Inventory (BAI) (48), which asks participants how much they have been bothered by a range of symptoms of anxiety (e.g., unable to relax, fear of worst happening, heart pounding/racing) during the past month. Responses were measured on a 4-point scale from (1) *not at all* to (4) *severely, it bothered me a lot* and averaged to create a composite anxiety score (α = 0.95).

*Substance Use*. To measure substance use, we administered the 16-item GAIN Substance Problem Scale (49), which asks participants when they last performed behaviors or experienced outcomes associated with problematic substance use (e.g., tried to hide that you were using alcohol or other drugs, unable to cut down on or stop using alcohol or other drugs). Responses were measured on a 6-point scale with the following options: (1) *never*, (2) *more than 12 months ago*, (3) *between 6 and 12 months ago*, (4) *between 3 and 5 months ago*, (5) *between 1 and 2 months ago*, and (6) *within the past month*. These items were averaged to create a composite substance use score (α = 0.94).

##### Social Media Use

Participants answered two items about their daily social media use: (1) “How many hours per day are you logged in on social media?” and, (2) “How many hours per day do you actively use social media?” By asking separate questions, our goal was to prompt participants to make a distinction between their time spent logged into social media and their hours of active social media use in an average day. Our interest was primarily in the latter item. Response options for both questions ranged from 0 to 24 h with 1 h increments.

We also measured active and passive social media use with a scale developed by Escobar-Viera et al. (29). Participants were asked “How often do you engage in the behaviors listed below while using any social media site?,” with four items assessing active social media use (*like/favorite/voting*; *share others' content—e.g., retweet, share posts or status updates*; *comment on or respond to someone else's content*; *post your own content—e.g., tweet, status update*) and three items assessing passive social media use (*read discussions*; *read comments/reviews*; *watch videos or view pictures*). Responses were measured on a 6-point scale with the following options: (0) *never*, (1) *less than once a week*, (2) *once a week*, (3) *2–6 times a week*, (4) *once a day*, and (5) *several times a day*. Composite scores were then calculated for active social media use (ASMU; α = 0.82) and passive social media use (PSMU; α = 0.74), with higher scores reflecting a higher frequency of each type of use.

##### Cyberbullying

CBV and CBP were once again measured using items adapted from previous research (44), with a few key modifications. First, whereas in Study 1, participants were asked about the frequency with which they had experienced or performed specific cyberbullying actions “in the last 2 months,” in Study 2, we asked how frequently they had experienced or performed specific cyberbullying actions “in your life.” We felt that assessing cyberbullying experiences across a broader time frame might capture greater variability in our adult sample. In light of this, we employed a 5-point response scale with the following options: *never, once, a few times, several times*, and *many times*. Second, we modified the wording of all CBV and CBP items so that they explicitly asked about cyberbullying experiences “on or using social media.” Finally, in Study 2, we used identical items to measure CBV and CBP, with the only difference being whether participants were instructed to “Please indicate the extent to which you have experienced each of the following in your life” (CBV) or to “Please indicate the extent to which you have performed each of the following in your life” (CBP). The specific items included: (1) someone posted mean or hurtful comments about me (CBV) / posted mean or hurtful comments about someone else (CBP); (2) someone posted a mean or hurtful picture of me (CBV) / posted a mean or hurtful picture of someone else (CBP); (3) someone posted a mean or hurtful video of me (CBV) / posted a mean or hurtful video of someone else (CBP); (4) someone created a mean or hurtful social media group or page about me (CBV) / created a mean or hurtful social media group or page about someone else (CBP); (5) someone spread rumors about me (CBV) / spread rumors about someone else (CBP); (6) someone threatened to hurt me (CBV) / threatened to hurt someone else (CBP); and (7) someone else pretended to be me to cause harm (CBV) / pretended to be someone else to cause harm (CBP). The items assessing CBV (7 items; α = 0.82) and CBP (7 items; α = 0.88) demonstrated good reliability and were thus averaged to create composite CBV and CBP variables. In contrast to Study 1, the composite CBV and CBP variables were only moderately positively correlated (*r* = 0.49, *p* < 0.001), which is consistent with previous research (13).

##### Attention Checks

Two attention checks were administered in the survey. Participants were instructed to “please select 3” on a multiple-choice item embedded within the SMU-UNS scale (see below) and participants were instructed to select “more than 12 months ago” on a filler item included in the substance use scale. Participants who failed both attention checks (*n* = 1) were excluded from analyses.

##### Exploratory Measures

We also included the following measures for exploratory purposes: (1) which of 14 different social media platforms participants currently use, with the option to select “other” and provide a text response to indicate use of a social media platform that did not appear on the list; (2) digital status seeking, assessed using four items from Nesi and Prinstein (50) (e.g., “I think it's important to have a lot of followers or friends on social media”), with responses indicated on a 5-point scale from *not at all true* to *extremely true*; (3) privacy preferences, assessed with two items about privacy settings on social media (e.g., “How do you control the privacy settings of your social media accounts?”), with five response options (*I adjust my privacy settings to control who has access to what I publish on my account*; *I am aware of different levels of privacy but don't really care about controlling them*; *I am not sure how I can control the privacy settings on my social media accounts*; *I don't use social media*; and *other*); (4) motives for social media use, assessed using the Scale of Motives for Using Social Networking Sites (SMU-SNS) (51), which measures nine potential motives (e.g., dating, new friendships, social connectedness, entertainment, self-expression, information-seeking) on a 7-point scale from *completely untrue* to *completely true*; and (5) impact of COVID-19, assessed by asking how much the threat of the coronavirus had negatively impacted 13 aspects of one's life (e.g., “your relationships,” “your physical health,” “your happiness and well-being,” “your degree of activity on social media”), as well as three items about COVID-19-related distress (e.g., “How stressful has the threat of the coronavirus been for you?”), with responses on a 5-point scale from *not at all* to *a great deal*. These exploratory measures were not included in any of the analyses reported below.

#### Analytic Strategy

Mirroring the analyses in Study 1, we investigated the extent to which degree of active social media use moderates the relation between mental health and cyberbullying through a series of multiple regression models. In each model, one mental health variable (i.e., depression, anxiety, substance use), one indicator of active social media use (i.e., ASMU, daily hours active on social media), a grouping variable for gender (coded: men = −1, women = 1), and all potential interactions were entered as predictors of either CBV or CBP, with age entered as a covariate. Missing data for age was deleted pairwise, with the sample size for specific analyses ranging from 481 to 482. Composite scores for the multi-item scales (i.e., depression, anxiety, substance use, ASMU, CBV, CBP) reflect the mean of available items for cases with item-level missing data. Continuous predictors were mean-centered prior to the calculation of interaction terms and significant interactions were probed at 1 *SD* below the mean, at the mean, and at 1 *SD* above the mean for a given moderator.

### Results

Descriptive statistics and bivariate correlations are reported in Table 5. Below, we summarize the primary results of the analyses with each mental health variable, ASMU, and gender as predictors of CBV and CBP. The main and interaction effects for all models tested, as well as the results of a parallel set of regression analyses with daily hours of active social media use as the social media use variable, are presented in Table 6.

**Table 5:** Study 2: descriptives and bivariate correlations for major study variables.

		***M***	***SD***	**1**	**2**	**3**	**4**	**5**	**6**	**7**	**8**	**9**	**10**
1	Depression	1.96	0.64	–									
2	Anxiety	1.65	0.62	0.70^***^	–								
3	Substance use	1.61	0.99	0.30^***^	0.35^***^	–							
4	Hrs/day logged into social media	11.30	8.89	0.03	0.06	0.07	–						
5	Hrs/day actively using social media	4.60	2.64	0.10*	0.06	0.09	0.40^***^	–					
6	Passive social media use	5.03	1.01	0.05	0.05	−0.02	0.20^***^	0.21^***^	–				
7	Active social media use	3.46	1.28	−0.03	0.00	0.05	0.15^**^	0.28^***^	0.28^***^	–			
8	Cyberbullying victimization	1.35	0.50	0.28^***^	0.29^***^	0.29^***^	0.07	0.11^*^	−0.01	0.20^***^	–		
9	Cyberbullying perpetration	1.16	0.43	0.16^***^	0.18^***^	0.32^***^	0.05	0.08	−0.02	0.13^**^	0.49^***^	–	
10	Age	31.03	11.57	−0.16^***^	−0.11^*^	0.03	−0.26^***^	−0.18^***^	−0.06	0.08	−0.08	−0.02	–
11	Gender	–	–	0.05	0.12^**^	−0.05	0.08	0.08	−0.03	0.11^*^	0.00	−0.11^*^	0.08

*Gender (men = −1, women = +1)*;

*** *p < 0.001*;

** *p < 0.01*;

* *p < 0.05*.

**Table 6:** Study 2: results of regression analyses.

	**Cyberbullying victimization**				**Cyberbullying perpetration**			
**Active social media use**	***b***	***SE***	***t***	***R*^**2**^**	***b***	***SE***	***t***	***R*^**2**^**
***Depression (N=482)***				0.13				0.10
Age	−0.00	0.00	−1.16		0.00	0.00	−0.02	
Depression	0.22^***^	0.03	6.44		0.13^***^	0.03	4.24	
Active SM use	0.09^***^	0.02	5.00		0.06^***^	0.02	3.76	
Gender	−0.02	0.02	−0.84		−0.06^***^	0.02	−3.39	
Depression × active SM use	0.05^+^	0.03	1.78		0.09^***^	0.02	4.04	
Depression × gender	−0.06^+^	0.03	−1.68		−0.05	0.03	−1.61	
Active SM use × gender	−0.01	0.02	−0.56		−0.02	0.02	−1.24	
Depression × active SM use × gender	−0.01	0.03	−0.55		−0.06^*^	0.02	−2.45	
***Anxiety (N=482)***				0.14				0.13
Age	−0.00	0.00	−1.31		0.00	0.00	0.01	
Anxiety	0.25^***^	0.04	6.94		0.16^***^	0.03	5.26	
Active SM use	0.09^***^	0.02	4.92		0.06^***^	0.02	3.96	
Gender	−0.03	0.02	−1.41		−0.07^***^	0.02	−3.92	
Anxiety × active SM use	0.05^*^	0.03	2.05		0.11^***^	0.02	4.73	
Anxiety × gender	−0.06^+^	0.04	−1.80		−0.09^**^	0.03	−3.08	
Active SM use × gender	−0.01	0.02	−0.72		−0.02	0.02	−1.57	
Anxiety × active SM use × gender	−0.02	0.03	−0.64		−0.07^**^	0.02	−3.03	
***Substance use (N=482)***				0.15				0.19
Age	−0.005^*^	0.00	−2.42		−0.00	0.00	−0.79	
Substance use	0.15^***^	0.02	6.68		0.14^***^	0.02	7.61	
Active social media use	0.08^***^	0.02	4.34		0.04^**^	0.01	3.10	
Gender	0.00	0.02	0.05		−0.05^*^	0.02	−2.55	
Substance use × active SM use	0.04^*^	0.02	2.50		0.05^***^	0.01	4.03	
Substance use × gender	−0.03	0.02	−1.40		−0.08^***^	0.02	−4.24	
Active SM use × gender	−0.00	0.02	−0.10		−0.01	0.01	−0.51	
Substance use × active SM use × gender	−0.03^+^	0.02	−1.68		−0.04^**^	0.01	−3.04	
**Daily hours of active SM use**								
***Depression (N=481)***				0.11				0.07
Age	−0.00	0.00	−0.76		0.00	0.00	0.36	
Depression	0.21^***^	0.04	6.07		0.11^***^	0.03	3.57	
Daily hours of active SM use	0.02^+^	0.01	1.89		0.01^+^	0.01	1.88	
Gender	−0.00	0.02	−0.17		−0.05^**^	0.02	−2.68	
Depression × daily hrs active SM use	0.03^*^	0.01	2.30		0.04^**^	0.01	3.12	
Depression × gender	−0.04	0.03	−1.28		−0.02	0.03	−0.82	
Daily hrs active SM use × gender	0.00	0.01	0.004		−0.01	0.01	−1.21	
Depression × daily hours active SM use × gender	−0.02^+^	0.01	−1.79		−0.02	0.01	−1.57	
***Anxiety (N=481)***				0.12				0.10
Age	−0.00	0.00	−0.94		0.00	0.00	0.46	
Anxiety	0.24^***^	0.04	6.49		0.15^***^	0.03	4.69	
Daily hours of active SM use	0.02^*^	0.01	2.39		0.02^**^	0.01	2.74	
Gender	−0.02	0.02	−0.99		−0.06^***^	0.02	−3.35	
Anxiety × daily hrs active SM use	0.05^***^	0.01	3.64		0.04^***^	0.01	3.50	
Anxiety × gender	−0.06	0.04	−1.63		−0.07^*^	0.03	−2.43	
Daily hrs active SM use × gender	−0.01	0.01	−0.86		−0.01^+^	0.01	−1.81	
Anxiety × daily hrs active SM use × gender	−0.02	0.01	−1.44		−0.04^**^	0.01	−3.09	
***Substance use (N=481)***				0.14				0.16
Age	−0.004^+^	0.00	−1.92		−0.00	0.00	−0.31	
Substance use	0.13^***^	0.02	5.62		0.13^***^	0.02	6.70	
Daily hours of active SM use	0.01	0.01	1.43		0.01^+^	0.01	1.68	
Gender	0.01	0.02	0.27		−0.04^*^	0.02	−2.31	
Substance use × daily hrs active SM use	0.03^***^	0.01	4.34		0.02^**^	0.01	3.26	
Substance use × gender	−0.03	0.02	−1.14		−0.07^***^	0.02	−3.44	
Daily hrs active SM use × gender	−0.00	0.01	−0.36		−0.01	0.01	−1.14	
Substance use × daily hrs active SM use × gender	−0.01	0.01	−1.54		−0.01^*^	0.01	−2.40	

*This table includes the unstandardized regression coefficients, associated standard errors and t-values, and overall model R^2^ values for each main regression analysis, organized by dependent variable (CBV or CBP). The top three panels report the results with active social media use scores (Active SM Use). The lower three panels report the results with number of daily hours of active social media use (Daily Hrs Active SM Use). Gender was coded: men = −1, women = 1*;

*** *p < 0.001*;

** *p < 0.01*;

* *p < 0.05*;

+ *p < 0.10*.

#### Depression

In the model predicting CBV from depression, ASMU, and gender, there was a marginally significant interaction between depression and ASMU, *b* = 0.05, *SE* = 0.03, *t*_(473)_ = 1.78, *p* = 0.075, and a trend toward an interaction between depression and gender, *b* = −0.06, *SE* = 0.03, *t*_(473)_ = −1.68, *p* = 0.094. The three-way interaction was not significant. Although they failed to meet the threshold for statistical significance, we saw value in probing the two-way interactions. Thus, we performed a regression analysis with depression, ASMU, gender, the Depression × ASMU interaction term, and the Depression × Gender interaction term entered as predictors of CBV, with age included as a covariate. The results indicated that the conditional effect of depression on CBV was stronger for men (than women) and for participants with relatively higher levels of ASMU.

In the model predicting CBP, there was a significant three-way interaction between depression, ASMU, and gender, *b* = −0.06, *SE* = 0.02, *t*_(473)_ = −2.45, *p* = 0.015, that was driven by a significant Depression × ASMU interaction that emerged for men only, *b* = 0.15, *F*_(1, 473)_ = 19.23, *p* < 0.0001. For men with relatively lower ASMU, there was no relation between depression and CBP. There was, however, a positive relation between depression and CBP at moderate, *b* = 0.17, *SE* = 0.04, *t*_(473)_ = 4.09, *p* = 0.0001, and relatively higher levels of ASMU, *b* = 0.36, *SE* = 0.07, *t*_(473)_ = 5.55, *p* < 0.0001. The Depression × ASMU interaction was not significant for women (*p* = 0.238), however, it is worth noting that the pattern of simple slopes revealed a similar trend. That is, whereas depression and CBP were unrelated at lower levels of ASMU, greater depression was a marginally significant predictor of greater CBP at moderate levels of ASMU, *b* = 0.07, *SE* = 0.04, *t*_(473)_ = 1.91, *p* = 0.057, and a significant predictor at higher levels of ASMU, *b* = 0.13, *SE* = 0.05, *t*_(473)_ = 2.45, *p* = 0.015. Notably, the strength of these relations was weaker than those observed for men.

#### Anxiety

In the model predicting CBV from anxiety, ASMU, and gender, a significant interaction between anxiety and ASMU, *b* = 0.05, *SE* = 0.03, *t*_(473)_ = 2.05, *p* = 0.041, and a marginally significant interaction between anxiety and gender, *b* = −0.06, *SE* = 0.04, *t*_(473)_ = −1.80, *p* = 0.072, emerged. In the absence of a significant three-way interaction, we probed the two-way interactions by testing a subsequent regression model with anxiety, ASMU, gender, the interaction between anxiety and ASMU, and the interaction between anxiety and gender entered as predictors of CBV, with age covaried. Results indicated that the conditional effect of anxiety on CBV was stronger for men than women and for participants with greater ASMU.

In the model predicting CBP from anxiety, ASMU, and gender, a significant three-way interaction emerged, *b* = −0.07, *SE* = 0.02, *t*_(473)_ = −3.03, *p* = 0.003, driven by a significant Anxiety × ASMU interaction, *b* = 0.18, *F*_(1, 473)_ = 23.81, *p* < 0.0001, for men only. For men, anxiety and CBP were unrelated at relatively lower levels of active social media use. Greater anxiety predicted greater CBP at moderate, *b* = 0.25, *SE* = 0.05, *t*_(473)_ = 5.43, *p* < 0.0001, and relatively higher levels of ASMU, *b* = 0.48, *SE* = 0.07, *t*_(473)_ = 6.81, *p* < 0.0001. Again, although the Anxiety × ASMU interaction was non-significant for women (*p* = 0.165), the pattern of simple slopes was suggestive of a similar trend. For women, anxiety and CBP were unrelated at lower levels of ASMU. At moderate levels of ASMU, greater anxiety was a marginally significant predictor of greater CBP, *b* = 0.07, *SE* = 0.04, *t*_(473)_ = 1.73, *p* = 0.085, and at higher levels of ASMU, greater anxiety was a significant predictor of greater CBP, *b* = 0.12, *SE* = 0.05, *t*_(473)_ = 2.30, *p* = 0.022. Again, the magnitude of this relation was considerably weaker than the relation observed for men.

#### Substance Use

In the model predicting CBV from substance use, ASMU, and gender, a trend toward a three-way interaction (that only approached marginal significance) emerged, *b* = −0.03, *SE* = 0.02, *t*_(473)_ = −1.68, *p* = 0.094, driven by a significant Substance Use × ASMU interaction, *b* = 0.07, *F*_(1, 473)_ = 7.53, *p* = 0.006, for men only. Across all levels of ASMU, substance use was positively correlated with CBV. The relation did, however, become significantly stronger with greater ASMU (−1 *SD*: *b* = 0.09, *SE* = 0.04, *t*_(473)_ = 2.07, *p* = 0.039; mean: *b* = 0.18, *SE* = 0.03, *t*_(473)_ = 5.57, *p* < 0.0001; +1 *SD*: *b* = 0.27, *SE* = 0.05, *t*_(473)_ = 5.75, *p* < 0.0001). The Substance Use × ASMU interaction was non-significant for women (*p* = 0.531). Across all levels of ASMU, greater substance use was associated with greater CPV in women, however, the simple slopes were suggestive of a trend that was similar to the pattern for men (−1 *SD*: *b* = 0.10, *SE* = 0.04, *t*_(473)_ = 2.26, *p* = 0.025; mean: *b* = 0.12, *SE* = 0.03, *t*_(473)_ = 3.84, *p* = 0.0001; +1 *SD*: *b* = 0.13, *SE* = 0.04, *t*_(473)_ = 3.60, *p* = 0.0004).

In the model predicting CBP from substance use, ASMU, and gender, a significant three-way interaction emerged, *b* = −0.04, *SE* = 0.01, *t*_(473)_ = −3.04, *p* = 0.003, driven by a significant Substance Use × ASMU interaction, *b* = 0.09, *F*_(1, 473)_ = 21.58, *p* < 0.0001, for men only. Across all levels of ASMU, substance use was positively correlated with CBP. The relation did, however, become significantly stronger with greater ASMU (−1 *SD*: *b* = 0.09, *SE* = 0.04, *t*_(473)_ = 2.58, *p* = 0.01; mean: *b* = 0.21, *SE* = 0.03, *t*_(473)_ = 8.16, *p* < 0.0001; +1 *SD*: *b* = 0.34, *SE* = 0.04, *t*_(473)_ = 8.86, *p* < 0.0001). The Substance Use × ASMU interaction was non-significant for women (*p* = 0.451). Greater substance use was associated with greater CPB, but only at moderate and higher levels of ASMU, and the strength of the substance use-CBP relation was considerably weaker than the relation observed for men (mean: *b* = 0.06, *SE* = 0.02, *t*_(473)_ = 2.45, *p* = 0.015; +1 *SD*: *b* = 0.08, *SE* = 0.03, *t*_(473)_ = 2.56, *p* = 0.011).

### Discussion

Study 2, designed to replicate Study 1 with psychometrically-validated, multi-item measures of depression, anxiety, substance use, and social media use, yielded a similar pattern of results. The associations between CBV and CBP, on one hand, and mental health, on the other, were significantly stronger among men, particularly at higher levels of active social media use. The congruence in results across studies speaks to the robustness of these effects and underscores the value of investigating cyberbullying in the context of social media use behavior and gender differences.

There was, however, one noteworthy difference in our Study 2 findings. In Study 1, depression and anxiety were uniformly uncorrelated with CBV and CBP among the women in our sample, regardless of their level of social media use. This finding stood in contrast to previous research documenting associations between cyberbullying and depression and between cyberbullying and anxiety in adults [e.g., (6, 9–11)]. In Study 2, more reliable positive correlations between cyberbullying and depression and anxiety emerged among women, albeit only at moderate and/or relatively higher levels of active social media use. One possibility is that the widely-used, clinically- and psychometrically-validated multi-item measures of depression and anxiety that we used in Study 2—the CES-D and Beck Anxiety Inventory—were much more effective at capturing variability in women's experiences of these psychological conditions. Yet, it is unclear why the single-item measures in Study 1 wouldn't be equally ineffective for the men in that sample. Moreover, even with the improved measures in Study 2, the relation between each indicator of mental health and both CBV and CBP was reliably weaker for women than for men. This elucidates a pivotal direction for future research.

## General Discussion

Across two studies, evidence of a stronger link between cyberbullying victimization (CBV) and cyberbullying perpetration (CBP), on one hand, and indicators of mental health—including depression, anxiety, and substance use—on the other, was found for adult men who reported more frequent and more active social media use. Crucially, however, this finding did not reliably emerge for adult women. In fact, the correlation between mental health and cyberbullying was strikingly weaker for women than men in Study 2 and absent from our results in Study 1. The pattern of gender differences obtained in the present research thus appears to contrast some of the existing work demonstrating a stronger association between cyberbullying and mental health for women (38) and girls (12, 13).

The present studies contribute to the relatively scarce literature on cyberbullying among adults in the general population. That is, the empirical literature on cyberbullying has focused almost exclusively on children and adolescents (4, 6), and the comparatively fewer studies of cyberbullying in adults have primarily examined college students and adults who experience cyberbullying in the workplace (or work-related contexts). Our hope is that the present research helps draw attention to this understudied phenomenon in adults and underscores the importance of better understanding the interrelations among cyberbullying, social media use, and mental health, particularly among men.

There are several limitations of the present research that warrant mention and highlight critical directions for future research. First, methodological characteristics of the research, including the cross-sectional design and reliance on self-report measures of mental health, social media use, and cyberbullying experiences, limit the generalizability of the findings. Future research with greater methodological diversity would offer complementary insights and strengthen the generalizability of the present findings. Particularly valuable insights, for instance, can be gained from longitudinal studies that track changes in cyberbullying, social media use, and mental health over time and by employing more objective indicators of social media use (e.g., number of actual social media posts made by a user during a designated time frame) instead of self-reported use.

A second limitation is the lack of diversity in our samples. Both samples were predominantly White (70.1% in Study 1, 66.0% in Study 2) and roughly half of the participants had at least a 4-year degree or higher (56.4% in Study 1, 48.8% in Study 2). Future research with more diverse adult samples is clearly needed to extend the present findings and because adults from marginalized populations may face an elevated risk of psychological disorders [e.g., (52)] and barriers to mental health treatment (53). For example, given recent research indicating that racial and ethnic minorities [e.g., (54)] are more likely to experience traditional bullying, it stands to reason that they may also be more susceptible to CBV. Although Kowalski et al. (55) found no significant differences in CBV between Black and White participants, they did, however, find that cyberbullying was more strongly linked to loneliness among Black compared to White participants. Thus, studies with more diverse adult samples are a vital direction for future research.

Finally, future research that speaks to the underlying causal relations among mental health, social media use, and cyberbullying would be particularly informative. Longitudinal (vs. cross-sectional) study designs may be especially beneficial for gaining insights about causal relations, given challenges associated with investigating cyberbullying and mental health experiences with experimental designs. Although these interrelations are likely somewhat bidirectional, research that sheds light on causal links may be instrumental in developing effective interventions.

## Data Availability Statement

The original contributions generated for the study are included in the article/supplementary material; further inquiries can be directed to the corresponding author.

## Ethics Statement

The studies involving human participants were reviewed and approved by the Arizona State University IRB. The patients/participants provided their informed consent to participate in this study.

## Author Contributions

KS devised the research question, assisted with Study 1 data collection, analyzed Study 1 data, co-wrote the initial draft of the manuscript, and assisted with manuscript revisions. SQ developed the materials for Study 2 and co-wrote and edited sections of the manuscript. BW analyzed Study 1 and Study 2 data and assisted with manuscript revisions. DH helped conceptualize the research question, collected and analyzed the data for Study 2, co-wrote the initial draft of the manuscript, and assisted with manuscript revisions. YS helped conceptualize and refine the research question and study design and helped prepare and provide critical feedback on the manuscript. All authors contributed to the article and approved the submitted version.

## Conflict of Interest

The authors declare that the research was conducted in the absence of any commercial or financial relationships that could be construed as a potential conflict of interest.
